# Assault and care characteristics of victims of sexual violence in eleven Médecins Sans Frontières programs in Africa. What about men and boys?

**DOI:** 10.1371/journal.pone.0237060

**Published:** 2020-08-04

**Authors:** Anaïs Broban, Rafael Van den Bergh, Wynne Russell, Guido Benedetti, Séverine Caluwaerts, Philip Owiti, Anthony Reid, Eva De Plecker

**Affiliations:** 1 Médecins Sans Frontières, Operational Center Brussels, Brussels, Belgium; 2 Médecins Sans Frontières, Operational Center Brussels, MSF Luxembourg (LuxOR), Luxembourg; 3 Médecins Sans Frontières, Consultant, Operational Center Brussels, Hobart, Australia; 4 Médecins Sans Frontières, Sexual and Reproductive Health unit, Operational Center Brussels, Brussels, Belgium; 5 International Union Against Tuberculosis and Lung Disease, Paris, France; University of Michigan, UNITED STATES

## Abstract

**Background:**

Often neglected, male-directed sexual violence (SV) has recently gained recognition as a significant issue. However, documentation of male SV patients, assaults and characteristics of presentation for care remains poor. Médecins Sans Frontières (MSF) systematically documented these in all victims admitted to eleven SV clinics in seven African countries between 2011 and 2017, providing a unique opportunity to describe SV patterns in male cases compared to females, according to age categories and contexts, thereby improving their access to SV care.

**Methods and findings:**

This was a multi-centric, cross-sectional study using routine program data. The study included 13550 SV cases, including 1009 males (7.5%). Proportions of males varied between programs and contexts, with the highest being recorded in migratory contexts (12.7%). Children (<13yrs) represented 34.3% of males. Different SV patterns appeared between younger and older males; while male children and adolescents were more often assaulted by known civilians, without physical violence, adult males more often endured violent assault, perpetrated by authority figures. Male patients presented more frequently to clinics providing integrated care (medical and psychological) for victims of violence (odds ratio 3.3, 95%CI 2.4–4.6), as compared to other types of clinics where SV disclosure upon admission was necessary. Males, particularly adults, were disproportionately more likely to suffer being compelled to rape (odds ratio 12.9, 95%CI 7.6–21.8).Retention in SV care was similar for males and females.

**Conclusions:**

Patterns of male-directed SV varied considerably according to contexts and age categories. A key finding was the importance of the clinic setup; integrated medical and SV clinics, where initial disclosure was not necessary to access care, appeared more likely to meet males’ needs, while accommodating females’ ones. All victims’ needs should be considered when planning SV services, with an emphasis on appropriately trained and trauma-informed medical staff, health promotion activities and increased psychosocial support.

## Introduction

Sexual violence (SV) is recognized as a worldwide problem and is defined by the World Health Organization as “*Any sexual act*, *attempt to obtain a sexual act*, *unwanted sexual comments or advances*, *or acts to traffic or otherwise directed against a person’s sexuality using coercion*, *by any person regardless of their relationship to the victim*, *in any setting*, *including but not limited to home and work*, *including SV acts committed against men and women*” [1].

Females remain most targeted for SV globally and represent the majority of survivors [2, 3].However, males can also be victims, both as children and as adults [4, 5]. Some studies suggest a prevalence of childhood sexual abuse in boys of up to 17% in non-conflict settings [6], and of SV against adult males as high as 24% to 33% in conflict-affected settings [7, 8]. In recent years, recognition of male-directed SV has grown steadily [9, 10] with the result that “there has never been a better time for male victims’ needs to be recognized as a priority public health issue worldwide” [11].

Male-directed SV experiences can vary considerably according to age groups and political and social contexts. Males are at risk of SV in all stages of life, as children [6], adolescents [12], or adults [13], with different patterns of abuse and perpetration at different ages. Childhood sexual abuse of boys is more commonly perpetrated by an older civilian outside the family, and outside the home [9]. During adolescence, boys are more likely to be assaulted by adults or older adolescents, including females, with motives more likely linked to social, financial or manipulative power and pressure [14]. After reaching adulthood, specific groups may be more at risk than others, such as men who have sex with men or prisoners [13]. In unstable contexts such as conflict zones or conditions of migration, men may also be selectively targeted: patterns may be more violent, committed by adult perpetrators (commonly military or paramilitary), and directed towards humiliation and power domination, including torture or stripping victims of their masculinity [15–17].

Nevertheless, studies of victims presenting for medical treatment for SV often show far lower proportions of males (usually less than 8%)than prevalence figures would suggest [18–21]. As for females, patterns commonly associated with SV renders disclosure difficult; these include stigma, shame and guilt, fear of not being believed or lose their social status, isolation, and ignorance (lack of words to describe the experience, uncertainty about which attitude to adopt). However, this underrepresentation of males is usually attributed to at least two broad factors. First, men and boys face a range of gender-role-specific barriers to reporting, including: 1. Cultural expectations of male invulnerability—either regarding the assault (“a man should be able to protect himself”) or its aftermath (“a man should be able to cope”); 2. Questioning of gender identity—they may feel feminized or homosexualized, particularly if they experienced an involuntary physical response to an assault (erection, ejaculation) [17]; 3. Fear of being labelled as homosexual regardless of any coercion involved, including in countries where homosexuality is criminalized; 4. Isolation—due to the silence and lack of awareness surrounding the issue, men and boys may even more believe that their experiences are unique [17, 22–24].

Second, the setup of SV services themselves may pose barriers to prevent male victims from coming forward, as male-directed SV is not always duly recognized by care providers and most services are tailored to meet females’ specific needs [25]; health promotion may be largely targeted towards women; clinics may be integrated into mother and child health units; and care plans may be more adapted to female victims’ needs [26]. Hence, males may have fewer access points for care [27]. While there have been calls to make SV care more accessible to males, there have been few meaningful results so far [4, 26, 27].

The small number of male patients in previous studies and the lack of interest in male-directed SV in previous decades have resulted in limited documentation of the characteristics, experiences or needs of male patients [26]. Few studies document the characteristics of SV assaults against males, and of their perpetrators; additionally, many studies have small sample sizes or are limited to certain contexts or age groups [9].A deeper understanding of the nature of sexual violence towards males and their perpetrators is needed for health actors to assess the true magnitude of the problem, and to improve case identification and appropriate care, especially in unstable contexts and/or among vulnerable populations [26].

Médecins Sans Frontières (MSF), Operational Centre Brussels (OCB), has for several years implemented a standardized package of care for victims of SV, both males and females, in a wide range of countries and contexts. In 2011, a database was introduced to systematically document all cases treated in MSF SV services. From 2011 to 2017, information on over 13,000 patients, including approximately 1,000 males, was recorded in eleven MSF SV services across seven African countries, providing a unique opportunity to examine the profiles and case management of male as compared to female SV victims on an unprecedentedly large scale, across age groups and political contexts.

In order to encourage presentation by male patients and the provision of adequate services, this study aimed to propose adaptations to SV clinics to better consider the needs of male and female victims, across different political contexts. As a prerequisite, we first documented characteristics of male-directed as compared to better-known female-directed SV in the specified MSF SV programs. Characteristics of male victims presenting for care were assessed across different assault types, circumstances, perpetrator profiles, and political contexts as compared to female victims and stratified by age categories.

A second part of the study focused on presentation for care of male and female victims of SV in the MSF programs. Specifically, we assessed differences between male and female SV experiences according to context, SV services setup, presentation for care, and treatment received, in the different age categories. Combining these two analyses allowed us to propose adaptations for more male-friendly services.

## Methods

### Design

This was a cross-sectional study using routine multi-centric program data.

### Setting

As a humanitarian medical organization, MSF works in various contexts, especially in emergency settings and among vulnerable populations. These contexts include conflict zones, post-conflict zones, migration zones—such as programs on well-identified migration routes and in areas of high population mobility—and stable urban or rural locations with underprivileged populations. In this range of settings, MSF has established specialized SV services to provide appropriate treatment for SV victims. These services have employed standardized guidelines in order to ensure that the same package of care was offered to every SV patient [19]. The services have offered three main types of access to SV care: through a SV clinic located within a Mother and Child Health unit (MCH); through a standalone SV clinic; or through a SV unit integrated within a clinic for care of victims of all types of violence.

This study used data from eleven different MSF programs with SV services, located in seven African countries in different contexts (Box 1). Due to security issues, the names of the countries and programs in this study are not disclosed. Four of the programs were located in country #1 (Central Africa, two in rural active conflict zones and two in post-conflict zones), two in country #2 (Central Africa, one urban active conflict zone, one post-conflict zone), one in country #3 (Central Africa, urban stable context in slum), one in country #4 (Southern Africa, migratory context in rural area), one in country #5 (Central Africa, urban active conflict zone), one in country #6 (Northern Africa, migratory context in large urban setting) and one in country #7 (Southern Africa, urban stable zone).

**Box 1. Definitions of program variables collected in Médecins Sans Frontières (MSF) Sexual Violence (SV) programs in Africa, as per MSF Operational Center Brussels (OCB) protocol, 2011 to 2017**

**Table:** 

**SV clinic setup**	**SV clinic—integrated into Mother and Child Health (MCH) unit:** SV services are physically integrated into MCH departments. SV patient first need to enter the MCH unit and has to disclose an SV event upfront to access consultation and services. Advertisement of the services is made in the community and at out-patient department, but most often focused on women’s health and directed towards women and MCH patients.
	**SV clinic—standalone:** SV services are not physically located in any other service and patients can access them without entering women-specific services. However, SV events must be disclosed to access consultation and services, and patients can be identified as having experienced an SV event, given that the clinic only focuses on this. Advertisement of the clinic is often directed to the community and focused on SV.
	**SV clinic—integrated into care for victims of violence:** SV services are physically integrated into services for care for all victims of violence including care for trauma, general health, and mental health. SV event disclosure is not necessary on admission, and consultations provide an opportunity for confidential disclosure, which can then lead to internal referral to the SV services. Advertisement of the clinic is directed to the community and not only focused on SV but rather on all types of violence.
**Context**	**Urban stable zone:** SV clinic set in stable area with no active conflict; services are directed towards the local population.
	**Conflict zone:** SV clinic set in active conflict zone, whether involving national army or militia.
	**Post-conflict zone:** SV clinic set in zone having undergone active conflict in previous years, and characterized by overall lack of infrastructure, social and legal framework.
	**Migratory zone:** SV clinic set in settings of displacement, such as known migratory routes or areas of high population mobility; services are directed towards a migrating population (refugees, asylum seekers, internally displaced persons, or economic migrants).

Most programs offered treatment for rape and sexual assault, including being compelled to rape (see Box 2 for definitions). However, one program (in country #6, migratory context in large urban setting) only treated cases involving coerced penetration (rape and compelled rape), due to organizational and patient intake issues.

**Box 2. Definitions of patient variables collected in MSF SV programs in Africa, 2011 to 2017**

**Table:** 

**Reason for delay** *(self-reported)*	**No delay:** all patients presenting within 72 hours after the (last) incident.
	**No access to healthcare**: patient prevented from accessing healthcare by perpetrator or family, an abduction situation, distance, no money to travel, security issues, or lack of family support (for children).
	**No knowledge about treatment**: patient did not know he/she should seek treatment and/or that there was free care available for his/her condition.
	**Afraid/Ashamed**: patient knew about available care, and was able to present to clinic, but was afraid or ashamed due to stigma, fear of not being believed or well treated, or unwillingness to disclose the assault.
	**Other**: any other reasons.
**Type of assault**	**Rape:** physically forced or otherwise coerced penetration—even if slight—of the vulva, anus or mouth using a penis, other body parts or an object
	**Compelled to rape:** one person is forced to rape another person against their will
	**Sexual assault:** any forced or otherwise coerced sexual act not including penetration.
**Perpetrator profile**	**Known civilian:** person known to the victim, and who does not belong to the below-mentioned categories
	**Unknown civilian:** a stranger who does not belong to the below-mentioned categories
	**Family member:** any member of the close or extended family, including life partner, cousin, uncle/aunt
	**Military:** a soldier or belonging to an armed group, including rebels and child soldiers
	**Organized gangs:** belonging to an organized gang, except known armed rebel groups
	**Policeman:** a member of the police force, either on or off duty at the time of the event
	**Institutional agent:** belonging to an institution like a school, orphanage, church, hospital, prison, administrative institution, and that has a power-related relationship with the patient (e.g. doctor-patient, guard-prisoner)
	**Other:** does not fall in the abovementioned categories
**Armed perpetrator**	The perpetrator used any kind of weapon: gun, knife, bottle, stick. [yes/no].
**Abduction**	The patient was captive for at least one night, either at his/her own house or any other place. [yes/no].
**Sexual violence context**	**Home:** the aggression happened in or around the patient’s home
	**Daily activity:** all normal daily life activities like walking in the streets, market selling, collecting firewood, workplace
	**Migratory:** aggression took place during the migration process or at a place of refuge
	**Abduction situation:** patient was abducted to another place where he/she was assaulted
	**Institutional:** aggression took place in an institution like a school, hospital, prison, church, others

### Study population

All cases recorded in the eleven MSF SV programs of interest between 2011 and 2017 presenting after any kind of SV were included in the study. Suspect cases, as well as those with gender information missing, were not included.

### Sexual violence care package

Patients presenting to any MSF SV clinic were received by a dedicated medical attendant. Though attendants in this study’s services were not initially trained for the specific needs of male patients regarding SV screening, communication, and disclosure, they did gain valuable experience over time in services where more male patients were presenting.

All patients in this study’s services were offered a standard, multi-sectoral package of care, which was standardized across all types of clinic setups, contexts, and only varied by gender in relation to contraception. This included prevention of infection (sexually transmitted diseases prophylaxis, HIV post-exposure prophylaxis if presenting within 72 hours, and vaccination for hepatitis B and tetanus); treatment of injuries; management of unwanted pregnancy (emergency contraception when presenting within 120 hours, or safe abortion support or referral, if requested); psychological support; legal support through provision of a medico-legal certificate; and referrals to partner organisations for social support. Three follow-up visits, where patients could receive follow-up vaccinations and psychosocial support, were recommended at one week, one month and three months after the first visit, although this schedule could be adapted to the patient’s needs and project’s context [19].

### Data variables, sources, definitions

We used the standardized SV database routinely implemented in all SV services of MSF OCB. Data were gathered for the period of operation of each service between 2011 and 2017. Data collection was conducted at field level by medical staff using a paper-based patient data form. Data entry and quality checks were handled in a patient-pseudonymized way by field data managers (no access to patient’s names, while tracking back data remained possible), using either Microsoft Excel or EpiData (v3.1) software. Data were then pooled, validated and anonymized at headquarters for compilation of the master study database. Countries were also pseudonymized [28].

At the patient level, variables included demographics (age, gender), presentation for care (time between occurrence of assault and presentation to care, reason for delay), care received (complete package of care or not, number of follow-up visits) and characteristics of sexual assault (type, associated violence/abduction, context of aggression, number and profile of perpetrator[s], whether perpetrator[s]were armed). The eleven programs were also categorized by clinic type and political context (for definitions of variables, see Boxes 1 and 2).

### Analysis and statistics

The data from the eleven programs, available in Excel or EpiData software, were pooled and statistical analysis was performed using Stata v13. Missing values were not inferred. Descriptive analysis of data was conducted, and differences between groups were assessed using Pearson’s X^2^ test (Chi-square). Crude and adjusted odds ratio (OR) analysis controlling for type of context (stable urban zone, conflict zone, post-conflict zone, migratory zone) were calculated. The level of significance was set at α = 5% and 95% confidence intervals (CI) were calculated. Cuzick’s test was used to test for trends across age categories [29], and the Kruskal-Wallis test for equality of distribution [30].

### Ethics

This study was based on anonymized MSF patient data, and all analysis was conducted without revealing the identity of any of the programs or countries represented. Consequently, this research fulfilled the exemption criteria set by the MSF Ethics Review Board for a posteriori analyses of routinely collected clinical data and did not require full MSF Ethics Review Board review. It was conducted with permission from the Médecins Sans Frontières Operational Centre Brussels (OCB) Medical Director.

## Results

A description of the sample population by age category, program and political context is presented in Table 1. There were 16715 cases recorded in the database and treated in MSF SV services. Of these, 3031 were excluded due to unclear SV event (unknown type of event n = 408, non-sexual aggression n = 640, suspected n = 1983), and 134 were excluded as gender information was not recorded.

**Table 1:** Characteristics of eleven MSF sexual violence programs in Africa, 2011 to 2017.

		N	Male		Female	
			N	(%)	N	(%)
*Total*		*13550*	*1009*	*(7*.*5)*	*12541*	*(92*.*5)*
**Age categories**		**13512**	**1004**		**12508**	
Children (0-12y)		2686	344	(12.8)	2342	(87.2)
Adolescents (13-19y)		4743	173	(3.7)	4570	(96.3)
Young adults (20–45)		5560	444	(8.0)	5116	(92.0)
Older adults (>45y)		523	43	(8.2)	480	(91.8)
**Type of context**		**13550**	**1009**		**12541**	
Stable urban zone		6764	543	(8.0)	6221	(92.0)
Conflict zone		3546	92	(2.6)	3454	(97.4)
Post-conflict zone		325	4	(1.2)	321	(98.8)
Migratory context		2915	370	(12.7)	2545	(87.3)
**SV clinic setup**		**13550**	**1009**		**12541**	
**Integrated into MCH unit**		**3127**	**75**	**(2.4)**	**3052**	**(97.6)**
	Conflict program #1	2291	62	(2.7)	2229	(97.3)
	Conflict program #2	511	9	(1.8)	502	(98.2)
	Post-conflict program #1	218	1	(0.5)	217	(99.5)
	Post-conflict program #2	41	3	(7.3)	38	(92.7)
	Post-conflict program #3	66	0	(0.0)	66	(100.0)
**Standalone**		**8176**	**596**	**(7.3)**	**7580**	**(92.7)**
	Conflict program #3	646	8	(1.2)	638	(98.8)
	Stable urban program #1	1431	98	(6.9)	1333	(93.1)
	Stable urban program #2	5333	445	(8.3)	4888	(91.7)
	Migratory program #1	766	45	(5.9)	721	(94.1)
**Integrated into care for victims of violence**		**2247**	**338**	**(15.0)**	**1909**	**(85.0)**
	Migratory program #2	2149	325	(15.1)	1824	(84.9)
	Conflict program #4	98	13	(13.3)	85	(86.7)

SV: Sexual violence. MCH: Mother and Child Health.

Of 13550 included SV cases, 1009 (7.5%) were males. Numbers and proportions of males varied according to political and program context. The highest proportion of male victims presented in stable urban zones (53.8% of total male presentations) and migratory contexts (36.7% of total male presentations). Programs clearly showed heterogeneity in the number of cases and the proportion of males, with the lowest proportion found in MCH units and the highest proportion found in SV clinics integrated into units offering care for victims of violence.

### Age

Proportionally, child cases were more likely to be males as compared to other age groups; conversely, adolescent cases were less likely to be males (Table 2).

**Table 2:** Differences in age categories between male and female victims of sexual violence in eleven MSF programs in Africa, 2011 to 2017.

		Total	Male		Female		Adjusted OR	
			N	(%)	N	(%)	OR [CI 95%]*	p-value
**Age categories**	*Total*	*13512*	*1004*		*12508*			
**Children (0-12y)**		2686	344	(34.3)	2342	(18.7)	2.3 [2.0–2.7]	<0.01
**Adolescents (13-19y)**		4743	173	(17.2)	4570	(36.6)	0.4 [0.3–0.4]	<0.01
**Young adults (20-45y)**		5560	444	(44.2)	5116	(40.9)	1.1 [1.0–1.3]	0.18^b^
**Older adults (>45y)**		523	43	(4.3)	480	(3.8)	1.5 [1.0–2.0]	0.03^a^

OR: Odds Ratio. CI: Confidence Interval

* Adjusted for the type of context (conflict zone, post-conflict zone, migratory zone or stable urban zone). Each category is analyzed as binary variable and compared to the sum of all other categories. Female is reference category.

^a^ Crude odds ratio for this category was not significant and significance is the effect of adjustment

^b^ Crude odds ratio for this category was significant and non-significance is the effect of adjustment

### Characteristics of assaults compared to females

Table 3 compares the characteristics of the assaults between males and females, adjusted for the context of the SV program. Males were less likely to have been raped than women, but were much more likely to have been compelled to rape another person. Sexual assaults towards males happened less often at home but more often in institutions such as schools or prisons/detention centers. Overall, males were less likely to endure associated violence during the assault; however among those who did, they were more likely than females to be beaten or mutilated. Moreover, in one program where additional data on associated violence was recorded, some males reported sexual torture and humiliating practices such as forced nudity (2.2% in men, versus 0.7% in women). Males were less likely to be assaulted by a family member, but more likely to suffer an assault by a known civilian or by police, and were more likely to be assaulted by multiple aggressors, compared to females.

**Table 3:** Differences in type of sexual assault and associated violence between cases against male and female in eleven MSF programs in Africa, 2011 to 2017.

		Total	Male		Female		Adjusted OR	
			N	(%)	N	(%)	OR [CI 95%]*	p-value
**Type of assault**	*Total*	*13550*	*1009*		*12541*			
**Rape**		13085	957	(94.8)	12128	(96.7)	0.6 [0.4–0.8]	<0.01
**Compelled to rape**		79	26	(2.6)	53	(0.4)	12.9 [7.6–21.8]	<0.01
**Sexual touching**		386	26	(2.6)	360	(2.9)	0.9 [0.6–1.3]	0.52
**Sexual violence context**	*Total*	*13125*	*963*		*12162*			
**Daily activities**		6755	456	(47.4)	6299	(51.8)	1.0 [0.9–1.1]	0.82^b^
**Home**		4501	298	(30.9)	4203	(34.6)	0.8 [0.7–0.9]	0.01
**Abduction situation**		780	53	(5.5)	727	(6.0)	0.7 [0.5–1.0]	0.03^a^
**During migration**		416	15	(1.6)	401	(3.3)	0.6 [0.4–1.0]	0.06^b^		
**Institution**			349	103	(10.7)	246	(2.0)	3.8 [2.9–4.9]	<0.01
**Other**		324	38	(3.9)	286	(2.3)	1.3 [0.9–1.8]	0.16^b^
**Abduction**	*Total*	*12696*	*966*		*11730*			
**Yes**		1210	81	(8.4)	1129	(9.6)	0.8 [0.7–1.1]	0.16
Associated violence**	*Total*	*13550*	*1009*		*12541*			
**No recorded associated violence**		10485	780	(77.3)	9705	(77.4)	0.8 [0.7–0.9]	0.01^a^
**Beaten**		2041	200	(19.8)	1841	(14.7)	1.4 [1.2–1.6]	<0.01
**Robbed**		598	22	(2.2)	576	(4.6)	0.7 [0.5–1.1]	0.14^b^
**Sexual exploitation**		411	15	(1.5)	396	(3.2)	1.1 [0.6–1.8]	0.81^b^
**Witnessed violence**		302	8	(0.8)	294	(2.3)	0.7 [0.4–1.5]	0.37^b^
**Raped in public**		197	14	(1.4)	183	(1.5)	1.5 [0.8–2.6]	0.18
**Mutilation**		65	10	(1.0)	55	(0.4)	2.3 [1.1–4.7]	0.02
**Destruction of goods**		61	3	(0.3)	58	(0.5)	1.7 [0.5–5.6]	0.38
**Forced labor**		26	1	(0.1)	25	(0.2)	1.0 [0.1–7.5]	0.99
**Other**		206	16	(1.6)	190	(1.5)	1.7 [1.0–2.9]	0.06
**Perpetrator’s profile**	*Total*	*12497*	*925*		*11572*			
**Known civilian**		4528	397	(42.9)	4131	(35.7)	1.5 [1.3–1.7]	<0.01
**Unknown civilian**		3018	248	(26.8)	2770	(23.9)	1.1 [0.9–1.3]	0.36^b^
**Family member**		2256	69	(7.5)	2187	(18.9)	0.3 [0.2–0.4]	<0.01
**Military**		1705	66	(7.1)	1639	(14.2)	1.3 [0.9–1.7]	0.17^b^
**Organized gangs**		351	35	(3.8)	316	(2.7)	0.9 [0.6–1.4]	0.74
**Policeman**		298	80	(8.7)	218	(1.9)	2.7 [2.0–3.6]	<0.01
**Institutional agent**		186	16	(1.7)	170	(1.5)	0.7 [0.4–1.2]	0.25
**Other**		155	14	(1.5)	141	(1.2)	0.7 [0.4–1.2]	0.23
**Number of perpetrator(s)**	*Total*	*11572*	*720*		*10852*			
**Single**		9290	561	(77.9)	8729	(80.4)	0.6 [0.5–0.7]	<0.01^a^
**Multiple**		2282	159	(22.1)	2123	(19.6)	1.6 [1.3–2.0]	<0.01^a^
**Armed perpetrator(s)**^$^	*Total*	*11385*	*732*		*10653*			
**Yes**		2956	124	(16.9)	2832	(26.6)	0.8 [0.6–1.0]	0.05^b^

OR: Odds Ratio. CI: Confidence Interval.

* Adjusted according to the type of context (conflict zone, post-conflict zone, migratory zone or stable urban zone). Each category is analyzed as binary variable and compared to the sum of all other categories. Female is reference category.

** Up to four associated violence recorded for each case.

^$^ Any kind of weapon.

^a^ Crude odds ratio for this category was not significant and significance is the effect of adjustment.

^b^ Crude odds ratio for this category was significant and non-significance is the effect of adjustment.

Table 4 compares these same characteristics among males, according to different age groups. More young patients presented in stable urban zones and more adult patients in conflict and migratory zones. The same analysis among females showed the same overall trends, but higher proportions than males presented in conflict zones, both in children and in adults, as opposed to migratory zones where female proportions were lower than those of males in all age groups (S1 Table).

**Table 4:** Differences in characteristics and circumstances of sexual assault among male victims of sexual violence according to different age categories in eleven MSF programs in Africa, 2011–2017.

		Total	Children (0-12y)		Adolescents (13-19y)		Young adults (20-45y)		Older adults (>45y)		Cuzick* trend test
			n	(%)	n	(%)	n	(%)	n	(%)	p-value
**Context**	*Total*	*1004*	*344*		*173*		*444*		*43*		
**Stable Urban zone**		542	277	(80.5)	117	(67.6)	143	(32.2)	5	(11.6)	<0.01
**Conflict zone**		92	10	(2.9)	13	(7.5)	63	(14.2)	6	(14.0)	<0.01
**Post-conflict zone**		4	2	(0.6)	2	(1.2)	0	(0.0)	0	(0.0)	0.15
**Migratory zone**		366	55	(16.0)	41	(23.7)	238	(53.6)	32	(74.4)	<0.01
**Type of assault**	*Total*	*1004*	*344*		*173*		*444*		*43*		
**Rape**		952	324	(94.2)	165	(95.4)	423	(95.3)	40	(93.0)	0.69
**Compelled to rape**		26	3	(0.9)	4	(2.3)	16	(3.6)	3	(7.0)	<0.01
**Sexual touching**		26	17	(4.9)	4	(2.3)	5	(1.1)	0	(0.0)	<0.01
**Sexual violence context**	*Total*	*958*	*332*		*167*		*419*		40		
**Daily activities**		456	164	(49.4)	89	(53.3)	190	(45.4)	13	(32.5)	0.06
**Home**		294	140	(42.2)	50	(29.9)	93	(22.2)	11	(27.5)	<0.01
**Abduction situation**		53	4	(1.2)	10	(6.0)	37	(8.8)	2	(5.0)	<0.01
**During migration**		15	0	(0.0)	2	(1.2)	12	(2.9)	1	(2.5)	<0.01
**Institution**		103	10	(3.0)	8	(4.8)	73	(17.4)	12	(30.0)	<0.01
**Other**		37	14	(4.2)	8	(4.8)	14	(3.3)	1	(2.5)	0.44
**Abduction**	*Total*	*961*	*323*		*165*		*430*		*43*		
**Yes**		81	6	(1.9)	15	(9.1)	55	(12.8)	5	(11.6)	<0.01
Associated violence**	*Total*	*1004*	*344*		*173*		*444*		*43*		
**No recorded associated violence**		775	329	(95.6)	150	(86.7)	279	(62.8)	17	(39.5)	<0.01
**Beaten**		200	11	(3.2)	21	(12.1)	144	(32.4)	24	(55.8)	<0.01
**Robbed**		22	0	(0.0)	4	(2.3)	17	(3.8)	1	(2.3)	<0.01
**Sexual exploitation**		15	4	(1.2)	0	(0.0)	11	(2.5)	0	(0.0)	0.25
**Witnessed violence**		8	0	(0.0)	0	(0.0)	6	(1.4)	2	(4.7)	<0.01
**Raped in public**		14	0	(0.0)	1	(0.6)	12	(2.7)	1	(2.3)	<0.01
**Mutilation**		10	0	(0.0)	1	(0.6)	9	(2.0)	0	(0.0)	0.02
**Destruction of goods**		3	0	(0.0)	0	(0.0)	2	(0.5)	1	(2.3)	0.04
**Forced labor**		1	0	(0.0)	0	(0.0)	1	(0.2)	0	(0.0)	0.40
**Other**		16	0	(0.0)	1	(0.6)	14	(3.2)	1	(2.3)	<0.01
**Perpetrator’s profile**	*Total*	*921*	*320*		*163*		*397*		*41*		
**Known civilian**		394	223	(69.7)	79	(48.4)	87	(21.9)	5	(12.2)	<0.01
**Unknown civilian**		248	43	(13.5)	45	(27.6)	147	(37.0)	13	(31.7)	<0.01
**Family member**		68	49	(15.3)	13	(8.0)	6	(1.5)	0	(0.0)	<0.01
**Military**		66	0	(0.0)	5	(3.1)	50	(12.6)	11	(26.8)	<0.01
**Organized gangs**		35	2	(0.6)	7	(4.3)	24	(6.1)	2	(4.9)	<0.01
**Policeman**		80	1	(0.3)	8	(4.9)	62	(15.6)	9	(22.0)	<0.01
**Institutional agent**		16	0	(0.0)	5	(3.1)	11	(2.8)	0	(0.0)	0.03
**Other**		14	2	(0.6)	1	(0.6)	10	(2.5)	1	(2.4)	0.03
**Number of perpetrator(s)**	*Total*	*718*	*310*		*140*		*247*		*21*		
**Single**		559	281	(90.7)	118	(84.3)	148	(59.9)	12	(57.1)	<0.01
**Multiple**		159	29	(9.3)	22	(15.7)	99	(40.1)	9	(42.9)	<0.01
**Armed perpetrator(s)**	*Total*	*730*	*309*		*141*		*258*		*22*		
**Yes**		124	10	(3.2)	27	(19.2)	76	(29.5)	11	(50.0)	<0.01

MCH: Mother and Child Health; SV: Sexual Violence.

* For categorical variables, each category was compared with all the other ones.

** Up to four recorded associated violence per case.

While the proportion of rape among males was roughly the same in all age categories, male children were more likely to suffer sexual assault (excluding coerced penetration), while adult males were significantly more likely to be compelled to rape. Younger patients were more often assaulted at home and by known civilians than older ones, who were more frequently assaulted during abduction, migration, or in institutions. Assaults against older males were more likely to be perpetrated by unknown civilians, the military, policemen, or organized gangs, and to be perpetrated by multiple armed assaulters. Most associated violence occurred against older patients; they were more likely to endure most types of violence. Although similar observations in relation to assault and perpetrator characteristics by age categories were made among female patients, trends for most of these variables appeared less pronounced than in male patients (S1 Table).

### Access to care and treatment characteristics

Table 5 shows that patients presenting to an integrated clinic for care of victims of violence, including SV, were 3.3 times more likely to be males, as opposed to patients presenting to MCH-associated or standalone SV services. While there was no statistical difference between males and females in the length of delay in presenting for care, males more often stated that they did not have access to treatment.

**Table 5:** Differences in presentation to care and in received treatment between male and female victims of sexual violence in eleven MSF sexual violence programs in Africa, 2011 to 2017.

		Total	Male		Female		Adjusted OR	
			N	(%)	N	(%)	OR [CI 95%]*	p-value
**SV clinic setup**	*Total*	*13550*	*1009*		*12541*			
**Integrated into MCH unit**		3127	75	(7.4)	3052	(24.4)	0.9 [0.5–1.5]	0.66^b^
**Standalone**		8176	596	(59.1)	7580	(60.4)	0.4 [0.3–0.5]	<0.01^a^
**Integrated into care for victims of violence**		2247	338	(33.5)	1909	(15.2)	3.3 [2.4–4.6]	<0.01
**Time to presentation for care**	*Total*	*13377*	*981*		*12396*			
**Less than 72 hours**		6162	424	(43.2)	5738	(46.3)	1.0 [0.9–1.1]	0.82
**72 hours– 1 month**		2668	184	(18.8)	2484	(20.0)	0.9 [0.8–1.1]	0.52
**1 month and above**		4547	373	(38.0)	4174	(33.7)	1.1 [0.9–1.2]	0.43^b^
Reason for delay**	*Total*	*12349*	*905*		*11444*			
**No delay**		6162	424	(46.9)	5738	(50.1)	0.9 [0.8–1.1]	0.36
**No access to healthcare**		1606	155	(17.1)	1451	(12.7)	1.4 [1.1–1.6]	0.01
**No knowledge about treatment**		1880	168	(18.6)	1712	(15.0)	1.2 [1.0–1.4]	0.14^b^
**Afraid/Ashamed**		1800	108	(11.9)	1692	(14.8)	0.8 [0.6–1.0]	0.05^b^
**Other**		901	50	(5.5)	851	(7.4)	0.7 [0.6–1.0]	0.06^b^
Total 1^st^ visit package of care provided***	*Total*	*12541*	*893*		*11648*			
**Yes**		4454	240	(26.9)	4214	(36.2)	0.8 [0.7–1.0]	0.03
**Follow-up**	*Total*	*13317*	*1003*		*12314*			
**At least one follow-up visit**		7519	542	(54.0)	6977	(56.7)	0.9 [0.8–1.0]	0.11
**Mean number of follow-up visits**		1.0	1.0		1.0		/	0.68^$^

OR: Odds Ratio. CI: Confidence Interval. MCH: Mother and Child Health; SV: Sexual Violence.

* Adjusted for the type of context (conflict zone, post-conflict zone, migratory zone or stable urban zone). Each category is analyzed as binary variable and compared to the sum of all other categories. Female is reference category.

** Patients presenting less than 72 hours after sexual violence are considered to have no delay in presentation.

*** Calculated from the different components of care provided, according to standard package of care. Care not necessary, refusal or non-availability, all resulted here in package of care not provided. Physical examination, as well as HIV prophylaxis, Sexual Transmittable Infections treatment, tetanus and hepatitis B vaccination and psychological consultation were taken into account in this variable.

^a^ Crude odds ratio for this category was not significant and significance is the effect of adjustment.

^b^ Crude odds ratio for this category was significant and non-significance is the effect of adjustment

^$^ Kruskal-Wallis test for equality of distribution.

Overall, the proportion of patients receiving the complete package of care was relatively low, and a difference between genders was noticed; this difference mostly came from only one program, and specifically involved vaccination access. Males adhered to care at the same rate as females, with no statistically significant difference in number of follow-up visits between genders.

Given that health-seeking behavior may be affected by the age of the victim, we analyzed male cases by age group for characteristics of presentation for care (Table 6). Within the male population, SV clinics integrated into MCH or into care services for victims of violence saw more adults, while stand-alone SV clinics saw more young men and boys. There was no significant age-related trend among patients presenting within 72 hours; however among those who delayed presentation for care, older patients tended to present even later than younger ones, with the greatest delays among men aged over 45. The reason for delay among younger patients was more likely to be no access to healthcare or fear/shame, while older patients were more likely to have no knowledge of SV care. Chances of receiving a full package of care, as well as the mean number of follow-up visits, increased with age; these results are in line with those among female patients (S2 Table).

**Table 6:** Differences in access to care and treatment received between age categories among male cases in eleven MSF sexual violence programs in Africa, 2011 to 2017.

		Total	Children (0-12y)		Adolescents (13-19y)		Young adults (20-45y)		Older adults (>45y)		Cuzick* trend test
			n	(%)	n	(%)	n	(%)	n	(%)	p-value
**SV clinic setup**	*Total*	*1004*	*344*		*173*		*444*		*43*		
**Integrated into MCH unit**		75	8	(2.3)	11	(6.4)	51	(11.5)	5	(11.6)	<0.01
**Standalone**		595	298	(86.6)	128	(74.0)	163	(36.7)	6	(14.0)	<0.01
**Integrated into care for victims of violence**		334	38	(11.1)	34	(19.6)	230	(51.8)	32	(74.4)	<0.01
**Time to presentation for care**	*Total*	*976*	*323*		*171*		*439*		*43*		
**Less than 72 hours**		422	139	(43.0)	67	(39.2)	203	(46.2)	13	(30.2)	0.86
**72 hours– 1 month**		184	97	(30.1)	28	(16.4)	56	(12.8)	3	(7.0)	<0.01
**1 month and above**		370	87	(26.9)	76	(44.4)	180	(41.0)	27	(62.8)	<0.01
Reason for delay**	*Total*	*900*	*304*		*152*		*404*		*40*		
**No delay**		422	139	(45.7)	67	(44.1)	203	(50.2)	13	(32.5)	0.70
**No access to healthcare**		155	73	(24.0)	23	(15.1)	54	(13.4)	5	(12.5)	<0.01
**No knowledge about treatment**		166	24	(7.9)	14	(9.2)	110	(27.2)	18	(45.0)	<0.01
**Afraid/Ashamed**		108	52	(17.1)	35	(23.0)	19	(4.7)	2	(5.0)	<0.01
**Other**		49	16	(5.3)	13	(8.6)	18	(4.5)	2	(5.0)	0.56
Total 1^st^ visit package of care provided***	*Total*	*888*	*320*		*156*		*375*		*37*		
**Yes**		240	45	(14.1)	36	(23.1)	147	(39.2)	12	(32.4)	<0.01
**Follow up**	*Total*	*998*	*342*		*173*		*440*		*43*		
**At least one follow-up visit**		*539*	*169*	(49.4)	*82*	(47.4)	*252*	(59.6)	*26*	(60.5)	<0.01
**Mean number of follow-up visits**		1.0	0.9		0.9		1.2		1.3		<0.01^$^

MCH: Mother and Child Health; SV: Sexual Violence

* For categorical variables, each category was analyzed as binary variable and compared to the sum of all other categories

** Up to four recorded associated violence per patient

*** Calculated from the different components of care provided, according to standard package of care. Care not necessary, refusal or non-availability, all resulted here in package of care not provided. Physical examination, as well as HIV prophylaxis, Sexual Transmittable Infections treatment, tetanus and hepatitis B vaccination and psychological consultation were taken into account in this variable.

^$^ Kruskal-Wallis test for equality of distribution

## Discussion

This study is unique in examining a large number of victims of SV of all ages, and includes a substantial sample of males (1009 males, 7.5% of all cases) permitting detailed comparisons of victims, perpetrators and assault characteristics and by clinic setup and treatment received, as well as gender comparisons, in all stages of life. The information was gathered from diverse contexts, clinic setups, and countries. Some of the findings of this study are in keeping with those of other studies; others, however, reveal new dimensions of the male—and female—experience of SV and of SV care. Such information is important to guide the development of SV care for both males and females.

### Age categories

The age distribution of SV victims showed a distinct difference between males and females: compared to other age groups, child cases were more likely to be males, while adolescents were more likely to be female. This is consistent with some existing literature [9, 31], though higher figures of adolescent male cases were expected [12, 32]. Although it is possible that uneven standardization of SV definitions among medical staff and communities may have caused misclassification of SV among adolescents [33], this finding highlights the importance of health promotion activities directed to adolescents, especially males, and of adolescent-friendly SV services, which are sometimes neglected.

### Patterns by political context

Overall, programs in stable urban and migratory contexts had the highest proportions of male presentations; all contexts, nevertheless, included at least one program reporting a male proportion of 5.9% or above. This finding is consistent with studies that show that all of these environments pose sexual danger for males, although sometimes linked to different ages [7, 8, 15, 16, 31, 34, 35].

Age patterns seemed to be associated with the political context in which the programs were situated. Most male cases in stable contexts were children or adolescents, and those in migratory contexts were usually adults. While some studies have shown a high prevalence of child sexual abuse in stable contexts [31, 32] and of adults in migratory settings [26, 34], this study is the first to show the age distribution across different contexts. Most male cases in migratory settings involved adults, which is consistent with the fact that a majority of migrants are adults [36]; but importantly, proportions of children and adolescents in migratory settings were not negligible, underlining the need for SV services in this context to be able to offer care to children as well.

Developing this capacity notably includes appropriate training for medical attendants to cover all ages of patients, as well as being sensitive to gender, in particular regarding psychological and psychosocial support. Patient trust in medical staff is key to successful treatment of the mental health consequences of abuse [37]; sensitization of medical staff to the types of SV trauma and their mental health consequences in all age categories will help patients gain trust towards the staff. Knowledge of the context and its associated patterns of SV will also help in tailoring the services to cope with the medical and psychological needs of the patients [26]. In addition, understanding of cultural and social dynamics regarding SV will also help in providing quality psychosocial care [38, 39].

In conflict zones, most male cases were adults as expected [7, 8, 17]. Though the number of male cases was low across the conflict programs, we suggest that this may be related to the generally more female-friendly services in conflict settings in the current sample of MSF programs, which may have discouraged men from seeking care.

Within each context, proportions of male cases often varied widely between different programs—in conflict contexts, for example, proportions varied between 1.8% (Conflict program #2) and 13.3% (Conflict program #4). It therefore appears necessary to look beyond the context in order to explain differences in proportions of males between different programs.

### Assaults and perpetrator characteristics in children and adolescents cases

Overall, sexual assault against children was usually perpetrated by single and unarmed assaulters, who were known to the child, and typically involved less associated violence. Compared to females, male children were less likely to be assaulted by a family member and at home, which is consistent with previous studies [9, 31, 40]. However, most male children were assaulted by known civilians; this includes community members, older children or teenagers. This finding is also consistent with a previous study in South Africa [14].

The most frequently reported type of sexual assault against children and adolescents appeared to be rape [9]. However, many children encountered sexual assault without rape, the impact of which should not be underestimated given the potential lifetime consequences for mental health [32, 41, 42]. Impacts of child sexual abuse have been showed to potentially differ from those experienced by girls particularly in relation to gender identity issues [43].

### Assaults and perpetrator characteristics in adult cases

While the analysis showed that males were overall slightly less subject to rape than females, they were disproportionately more likely to be compelled to rape someone else; adults were clearly more affected. The phenomenon of compelled rape has been reported in a range of contexts, including migratory and conflict contexts [15, 22]; however almost no documentation exists on the prevalence or psychological consequences of this form of SV [17, 27], both for the person compelled to rape and the person raped, which can sometimes be closely linked one to another. These results highlight the need for further studies, including qualitative investigations, to develop appropriate medical and legal responses.

Overall, patterns of SV in adult males appeared to be more violent than among children and adolescents, with most perpetrators being unrelated to the victim and more often having a power-related relationship with the victim. This conclusion seems to be supported by every finding in this paper: adult males were more often abducted, assaulted by multiple and armed perpetrators, and/or assaulted by representatives of authorities or armed groups, and endured more associated violence of different kinds. Similar trends appeared among female cases, though overall to a lesser extent than among males (S1 Table).

These findings are consistent with previously described patterns of SV among male adults in conflict or migratory zones [34, 44, 45]–perhaps not unsurprisingly, as a high proportion of male adults in the sample came from migratory zones. However, literature on this subject remains scarce, especially regarding migratory settings, and should be further developed.

In one of the MSF programs, information was collected on sexual torture and humiliating procedures, such as forced nudity. These aspects are scarcely described in the literature, although an “open secret”[46], and the associated dynamics and psychological consequences are not clearly described or understood. Despite a growing focus on documenting sexual torture and humiliation in men [15, 16, 47, 48], there is a need for further studies exploring this finding, both quantitative and qualitative.

### Access to health care

Beyond the political context, the proportions of males presenting to different SV clinics point to clinic setup as a key factor. Overall, patients presenting at clinics providing integrated care (medical and psychological) for victims of violence, which did not require upfront disclosure of SV, were more likely to be male, as compared to those attending stand-alone SV clinics or clinics integrated into Maternal and Child Health units. Integrated clinics appear to address two of the main factors identified by previous studies as linked to male victims’ low access to care overall: orientation for care, and reluctance to disclose [45].

Indeed, the results suggest that orientation for care for males is improved in clinics for victims of violence; these clinics allow males to present through a gender-neutral entry door and to avoid disclosure on admission. It is therefore important to provide such an access route while organizing clinic activities, in order to better adapt to the needs of all genders and ages.

Meanwhile, the study underlines that each population has different needs, all of which may not be satisfied by a single access route. For instance, male children and adolescents presented to SV stand-alone clinics more often than adult males, which may relate to parents bringing them to the clinic versus adults seeking care on their own. More women also presented to clinics integrated into MCH services. Notably, sensitization of medical staff in all services, including child- and women-related services and general out-patient departments, will help facilitate multiple access routes in order to reach a maximum number of victims.

Moreover, reluctance to disclose can be addressed by providing a confidential environment and developing patient trust in the service. Anecdotally, though medical staff in the services surveyed had not initially received specific training for male-directed SV and recognizing associated signs, skills were developed over time notably in clinics integrated into care for victims of violence where more adult males were presenting, thus encouraging more disclosures. This highlights a need for strong staff education and sensitization around male-directed SV [25, 49, 50]. The need is especially important in contexts where integrated services for victims of violence are not available.

Though both sexes seemed to face barriers to disclosure due to fear or shame, it is encouraging, given the strong stigma around the experience of male-directed SV, that less than 5% of the study’s adult males (>20y) said that they had delayed seeking care due to stigma. However, the study does not capture victims who were too afraid or ashamed to seek care at all. Moreover, having no knowledge of treatment represented the first reason for delay among patients presenting late, both males and females (Table 6 and S2 Table), reaching 45.0% among older male patients. This points to the importance of awareness-raising and de-stigmatization campaigns around SV, especially male-directed SV, addressing not only victims themselves, but also communities as a whole [45].

Furthermore, the fact that younger patients presented earlier as compared to older patients may reflect the role of parents or guardians in supporting children and adolescents to overcome barriers to disclosure and to care-seeking [17, 39].

### Treatment received

While the total number of patients receiving the full package of care appears relatively low, the variable did not account for specific situations when some of the care was not needed (such as already completed vaccination schedules), when regulatory issues rendered access to some clinical supplies difficult, or when stock ruptures of clinical supplies occurred. Some patients also may have declined certain aspects of the care package offered, such as HIV prophylaxis, vaccination or physical examination. For instance, in one of the study’s countries, where over 10% of patients were male, vaccines were under restricted access and could not be provided by MSF but only by government facilities. The specific profile of male-directed sexual assaults, which in this context were more likely to have been perpetrated by authority figures, may have possibly impacted genders differentially with fewer males going on to be vaccinated in government facilities. This low rate could also reflect a need for additional training of medical staff; however, the design of this retrospective routine data study does not allow us to know the exact reasons for the low full package delivery rate, especially in males. An additional focused study would be needed in order to determine further steps to address these issues.

There was no difference in the percentage of patients who came back for at least one follow-up visits between males and females, nor in the number of follow-up visits received, which shows that once enrolled in the program, males adhered to care at the same rate as females.

Our findings also suggest that all SV services need to be capable of managing child victims as well as adolescents, in addition to adults. The finding that younger patients, both males and females, came back less consistently to follow-up visits, compared to older patients, may be related to children’s dependence on caregivers as well as cultural narratives downplaying the impact of child sexual abuse as compared to adults [9]. As most young victims knew their perpetrators, informal mediation sought by some families with the perpetrator(s) could also have influenced on care discontinuation; this remains to be explored in more details.

Meanwhile, older patients’ willingness to return for psychological support and consultations may be linked to their higher incidence of more violent and stigmatizing experiences, which should be taken into account operationally by increasing psychosocial support capacity for this population.

### Strengths and limitations of the study

There are a number of strengths to this study. Standardized data collection and definitions (as in Boxes 1 and 2) were used across all the settings, giving validity and consistency to the data; moreover, data cleaning of all datasets at headquarters level ensured high quality and comparability across programs. The sample was very large and was the first to gather such a number of male SV victims. The population also represented diverse contexts, countries and cultures in Africa, which gave a unique overview of male-directed SV on the continent.

However, there were some limitations. The heterogeneity of the sample, while bringing a valuable overview of the whole picture, did not allow for a precise accounting of the impact of local contexts—cultural, political, military, etc.—thereby masking diversity in profiles and assault characteristics, as well as cultural dynamics and stigmas around perceptions of male-directed SV. The study also lacked the ability to analyze the gender and age of SV perpetrators. Moreover, appropriate attention was not always given to the needs of male victims of SV when establishing MSF OCB SV clinics, especially in conflict settings, which may explain the lower male presentation than expected in that context. Finally, as this study focused on patients who attended the SV clinics, there was a clear selection bias in the sample and we missed all victims who were unwilling or unable to access a clinic out of fear, stigma, or other reasons.

### Implications

There are some clear operational implications for quality of care which arise from the study. First, the setup of entry to SV services plays a crucial role in encouraging male patient intake, with male victims more strongly represented at clinics where SV disclosure is not an entry criterion; SV care providers are therefore encouraged to offer such an access route to favor male patients’ presentation for care.

Second, males’ needs and health-seeking behavior must be acknowledged as different from females’ and should trigger adapted services, such as additional training of medical attendants to receive all age categories of male victims, especially but not exclusively in conflict and migratory contexts. Trained staffs should include SV attendants, as well as attendants of other services (including out-patient department) in order to better recognize the signs of SV and encourage disclosure.

Third, improving the quality of care for male patients implies developing psychological care. Trauma-informed psychosocial services should be equipped to address the particular needs and experiences of male survivors, including the impacts of lesser-known types of SV such as compelled rape and humiliating assault, or child sexual abuse of boys.

Moreover, a deep understanding of each context, culture and local SV dynamics would help clinics frame and adapt the best SV services to fit the needs of male and female victims. This should include future qualitative research into the social norms associated with sexual violence towards men across various contexts where sexual violence care programs are being set up. Finally, health promotion activities adapted to each age category are important and should be increased to make victims, both males and females, aware of available treatment and to diminish the level of stigma associated with SV.

In addition to implications for quality of care, in-depth knowledge of SV male victims’ profiles, cultural context and local SV dynamics can also have broader implications for all field actors, including social actors or legal and human rights activists, in order to trigger actions addressing this issue. For example, the fact that an important part of adult male victims of SV were assaulted by authority representatives suggests a need for legal protection and support, including data confidentiality.

Finally, further research into male-directed SV, both quantitative and qualitative, continues to be necessary in order to gain a deeper understanding of the impacts associated with male-directed SV, including specific feminization and homosexualization dynamics, the psychological consequences of the different SV events, gender-specific dynamics across contexts and cultures, as well as associated health-seeking behavior. Further studies on sexual torture and humiliation would also assist services in developing appropriate medical and psychosocial care for male survivors [25, 47, 48]. All this knowledge will enhance trauma-informed care and will be crucial to adapting SV services to males’ needs.

## Conclusion

This study uniquely describes characteristics of victims of sexual assaults, types of assault, perpetrators, and access to care among a large number of male-directed SV cases in Africa. Substantially different patterns of sexual assaults emerged when males were compared to females, between different age categories and different contexts. The study suggests ways of adapting SV services to match these different patterns and ages, using knowledge of local contexts and SV dynamics.

A key finding was that the setup of entry into SV services was a crucial factor in encouraging more males, particularly adults, to access SV care, as disclosure on admission may discourage men’s access. However once disclosed, adherence to care appeared to be similar between genders. SV clinics are encouraged to provide an access route without upfront disclosure, possibly among multiple access routes. Appropriately trained medical staff, increased psychosocial support in clinics, along with health promotion activities in the community, may also improve male patients’ willingness to disclose and access to care. Moreover, health promotion activities tailored to male children, adolescents and adult SV victims will help raise awareness of this issue and ensure that care services better reach all SV victims.

Further studies are required to better explore the needs of male victims of SV, and this knowledge be used to adapt SV care for both males and females, of all ages.

## Supporting information

S1 Table Differences in characteristics and circumstances of sexual assault among female victims of sexual violence according to different age categories in eleven MSF programs in Africa, 2011–2017. (DOCX)

S2 Table Differences in access to care and treatment received between age categories among female patients in eleven MSF programs in Africa, 2011 to 2017. (DOCX)

S3 Table Completed STROBE checklist for cross-sectional studies and analysis plan information. This checklist was elaborated using formal items recommended for cross-sectional studies from STROBE statement (https://www.strobe-statement.org).
(DOC)
