# Correction: Assault and care characteristics of victims of sexual violence in eleven Médecins Sans Frontières programs in Africa. What about men and boys?

**DOI:** 10.1371/journal.pone.0299409

**Published:** 2024-02-20

**Authors:** Anaïs Broban, Rafael Van den Bergh, Wynne Russell, Guido Benedetti, Séverine Caluwaerts, Philip Owiti, Anthony Reid, Eva De Plecker

There are errors in the Funding and Competing Interests statements. The correct statements are:

**Funding**: Publication was supported by the Structured Operational Research and Training Initiative (SORT IT), a global partnership led by the Special Program for Research and Training in Tropical Diseases at the World Health Organization (WHO/TDR). Médecins sans Frontières (MSF) provided support in the form of salaries for AB, RVdB, GB, SC, AR, and EDP. The specific roles of these authors are articulated in the ‘author contributions’ section. MSF programmatic funding covered all other costs associated with the study. The funder otherwise was not involved in study design, data collection and analysis, decision to publish, or preparation of the manuscript.

**Competing Interests**: The authors have read the journal’s policy and have the following competing interests to declare: Médecins sans Frontières (MSF) provided support in the form of salaries for AB, RVdB, GB, SC, AR, and EDP. This does not alter our adherence to *PLOS ONE* policies on sharing data and materials. There are no patents, products in development, or marketed products associated with this research to declare.
