# Supplementary material for: Assault and care characteristics of victims of sexual violence in eleven Médecins Sans Frontières programs in Africa. What about men and boys?
Source: PLoS One. 2020 Aug 4;15(8):e0237060. doi: 10.1371/journal.pone.0237060 (PMC7402504; doi:10.1371/journal.pone.0237060)
Supplement: S1 Table — (DOCX) [file pone.0237060.s001.docx]

**S1 Table.** **Differences in characteristics and circumstances of sexual assault among female victims of sexual violence according to different age categories in eleven MSF programs in Africa, 2011-2017**

|  | | | Total | | | | Children  (0-12y) | | | | | | | | | Adolescents  (13-19y) | | | | | | | | | Young adults  (20-45y) | | | | | | | | | Older adults  (>45y) | | | | | | | | | Cuzick^*^  trend test | | | |  |  |  |
| --- | --- | --- | --- | --- | --- | --- | --- | --- | --- | --- | --- | --- | --- | --- | --- | --- | --- | --- | --- | --- | --- | --- | --- | --- | --- | --- | --- | --- | --- | --- | --- | --- | --- | --- | --- | --- | --- | --- | --- | --- | --- | --- | --- | --- | --- | --- | --- | --- | --- |
|  | | |  |  |  |  | n | | | | (%) | | | | | n | | | | (%) | | | | | n | | | | (%) | | | | | n | | | | (%) | | | | | p-value | | | |  |  |  |
| Context | | *Total* | | | | *12508* | | | | *2342* | | | | |  | | | | 4570 | | | | |  | | | | 5116 | | | | |  | | | | 480 | | | | |  | | | |  | | | |
| Stable Urban zone | | | 6221 | | | | 1771 | | | | | (75.6) | | | | 2959 | | | | | (64.7) | | | | 1452 | | | | | (28.4) | | | | 39 | | | | | (8.2) | | | | <0.01 | | | |  |  |  |
| Conflict zone | | | 3450 | | | | 244 | | | | | (10.4) | | | | 951 | | | | | (20.8) | | | | 1939 | | | | | (37.9) | | | | 316 | | | | | (65.8) | | | | <0.01 | | | |  |  |  |
| Post-conflict zone | | | 318 | | | | 94 | | | | | (4.0) | | | | 196 | | | | | (4.3) | | | | 26 | | | | | (0.5) | | | | 2 | | | | | (0.4) | | | | <0.01 | | | |  |  |  |
| Migratory zone | | | 2519 | | | | 233 | | | | | (10.0) | | | | 464 | | | | | (10.2) | | | | 1699 | | | | | (33.2) | | | | 123 | | | | | (25.6) | | | | <0.01 | | | |  |  |  |
| Type of assault | *Total* | | *12508* | | | | *2342* | | | | |  | | | | *4570* | | | | |  | | | | *5116* | | | | |  | | | | *480* | | | | |  | | | |  | | | |  |  |  |
| Rape | | | 12095 | | | | 2111 | | | | | (90.1) | | | | 4454 | | | | | (97.5) | | | | 5063 | | | | | (99.0) | | | | 467 | | | | | (97.3) | | | | <0.01 | | | |  |  |  |
| Compelled to rape | | | 53 | | | | 7 | | | | | (0.3) | | | | 16 | | | | | (0.3) | | | | 26 | | | | | (0.5) | | | | 4 | | | | | (0.8) | | | | 0.06 | | | |  |  |  |
| Sexual touching | | | 360 | | | | 224 | | | | | (9.6) | | | | 100 | | | | | (2.2) | | | | 27 | | | | | (0.5) | | | | 9 | | | | | (1.9) | | | | <0.01 | | | |  |  |  |
| Sexual violence context | | *Total* | *12133* | | | | *2253* | | | | |  | | | | *4478* | | | | |  | | | | *4932* | | | | |  | | | | *470* | | | | |  | | | |  | | | |  |  |  |
| Daily activities | | | 6290 | | | | 987 | | | | | (43.8) | | | | 2601 | | | | | (59.1) | | | | 2491 | | | | | (50.5) | | | | 211 | | | | | (44.9) | | | | <0.01 | | | |  |  |  |
| Home | | | 4189 | | | | 1117 | | | | | (49.6) | | | | 1348 | | | | | (30.1) | | | | 1569 | | | | | (31.8) | | | | 155 | | | | | (33.0) | | | | 0.26 | | | |  |  |  |
| Abduction situation | | | 726 | | | | 68 | | | | | (3.0) | | | | 308 | | | | | (6.9) | | | | 333 | | | | | (6.7) | | | | 17 | | | | | (3.6) | | | | <0.01 | | | |  |  |  |
| During migration | | | 400 | | | | 13 | | | | | (0.6) | | | | 76 | | | | | (1.7) | | | | 238 | | | | | (4.8) | | | | 73 | | | | | (15.5) | | | | <0.01 | | | |  |  |  |
| Institution | | | 245 | | | | 32 | | | | | (1.4) | | | | 36 | | | | | (0.8) | | | | 170 | | | | | (3.5) | | | | 7 | | | | | (1.5) | | | | <0.01 | | | |  |  |  |
| Other | | | 283 | | | | 36 | | | | | (1.6) | | | | 109 | | | | | (2.4) | | | | 131 | | | | | (2.7) | | | | 7 | | | | | (1.5) | | | | 0.07 | | | |  |  |  |
| Abduction | | *Total* | *11698* | | | | *2121* | | | | |  | | | | *4304* | | | | |  | | | | *4834* | | | | |  | | | | *439* | | | | |  | | | |  | | | |  |  |  |
| Yes | | | 1128 | | | | 97 | | | | | (4.6) | | | | 492 | | | | | (11.4) | | | | 504 | | | | | (10.4) | | | | 35 | | | | | (8.0) | | | | <0.01 | | | |  |  |  |
| Perpetrator’s profile | | *Total* | | *11543* | | | | *2149* | | | | |  | | | | *4348* | | | | |  | | | | *4603* | | | | |  | | | | *443* | | | | |  | | | |  | | | |  |  |
| Known civilian | | | 4125 | | | | 1262 | | | | | (58.7) | | | | 1743 | | | | | (40.1) | | | | 1075 | | | | | (23.3) | | | | 45 | | | | | (10.1) | | | | <0.01 | | | |  |  |  |
| Unknown civilian | | | 2761 | | | | 277 | | | | | (12.9) | | | | 823 | | | | | (18.9) | | | | 1534 | | | | | (33.3) | | | | 127 | | | | | (28.7) | | | | <0.01 | | | |  |  |  |
| Family member | | | 2187 | | | | 562 | | | | | (26.1) | | | | 1286 | | | | | (29.6) | | | | 327 | | | | | (7.1) | | | | 12 | | | | | (2.7) | | | | <0.01 | | | |  |  |  |
| Military | | | 1634 | | | | 22 | | | | | (1.0) | | | | 310 | | | | | (7.1) | | | | 1080 | | | | | (23.5) | | | | 222 | | | | | (50.1) | | | | <0.01 | | | |  |  |  |
| Organized gangs | | | 311 | | | | 2 | | | | | (0.1) | | | | 78 | | | | | (1.8) | | | | 220 | | | | | (4.8) | | | | 11 | | | | | (2.5) | | | | <0.01 | | | |  |  |  |
| Policeman | | | 216 | | | | 2 | | | | | (0.1) | | | | 23 | | | | | (0.5) | | | | 182 | | | | | (4.0) | | | | 9 | | | | | (2.0) | | | | <0.01 | | | |  |  |  |
| Institutional agent | | | 169 | | | | 8 | | | | | (0.4) | | | | 60 | | | | | (1.4) | | | | 94 | | | | | (2.0) | | | | 7 | | | | | (1.6) | | | | <0.01 | | | |  |  |  |
| Other | | | 140 | | | | 14 | | | | | (0.7) | | | | 25 | | | | | (0.6) | | | | 91 | | | | | (2.0) | | | | 10 | | | | | (2.3) | | | | <0.01 | | | |  |  |  |
| Associated violence** | | *Total* | *12508* | | | | *2342* | | | | |  | | | | *4570* | | | | |  | | | | *5116* | | | | |  | | | | *480* | | | | |  | | | |  | | | |  |  |  |
| No recorded associated violence | | | 9686 | | | | 2209 | | | | | (94.3) | | | | 3958 | | | | | (86.6) | | | | 3259 | | | | | (63.7) | | | | 260 | | | | | (54.2) | | | | <0.01 | | | |  |  |  |
| Beaten | | | 1829 | | | | 70 | | | | | (3.0) | | | | 377 | | | | | (8.3) | | | | 1270 | | | | | (24.8) | | | | 112 | | | | | (23.3) | | | | <0.01 | | | |  |  |  |
| Robbed | | | 576 | | | | 7 | | | | | (0.3) | | | | 95 | | | | | (2.1) | | | | 419 | | | | | (8.2) | | | | 55 | | | | | (11.5) | | | | <0.01 | | | |  |  |  |
| Sexual exploitation | | | 395 | | | | 53 | | | | | (2.3) | | | | 129 | | | | | (2.8) | | | | 187 | | | | | (3.7) | | | | 26 | | | | | (5.4) | | | | <0.01 | | | |  |  |  |
| Witnessed violence | | | 293 | | | | 8 | | | | | (0.3) | | | | 67 | | | | | (1.5) | | | | 169 | | | | | (3.3) | | | | 49 | | | | | (10.2) | | | | <0.01 | | | |  |  |  |
| Raped in public | | | 181 | | | | 1 | | | | | (0.1) | | | | 34 | | | | | (0.7) | | | | 121 | | | | | (2.4) | | | | 25 | | | | | (5.2) | | | | <0.01 | | | |  |  |  |
| Mutilation | | | 55 | | | | 7 | | | | | (0.3) | | | | 15 | | | | | (0.3) | | | | 31 | | | | | (0.6) | | | | 2 | | | | | (0.4) | | | | 0.05 | | | |  |  |  |
| Destruction of goods | | | 58 | | | | 0 | | | | | (0.0) | | | | 13 | | | | | (0.3) | | | | 44 | | | | | (0.9) | | | | 1 | | | | | (0.2) | | | | <0.01 | | | |  |  |  |
| Forced labor | | | 25 | | | | 0 | | | | | (0.0) | | | | 10 | | | | | (0.2) | | | | 14 | | | | | (0.3) | | | | 1 | | | | | (0.2) | | | | 0.04 | | | |  |  |  |
| Other | | | 190 | | | | 14 | | | | | (0.6) | | | | 56 | | | | | (1.2) | | | | 101 | | | | | (2.0) | | | | 19 | | | | | (4.0) | | | | <0.01 | | | |  |  |  |
| Number of perpetrator(s) | | *Total* | | | *10836* | | | | *2166* | | | | |  | | | | *4242* | | | | |  | | | | *4021* | | | | |  | | | | *407* | | | | |  | | | |  | | | |  |
| Single | | | 8719 | | | | 2046 | | | | | (95.5) | | | | 3783 | | | | | (89.2) | | | | 2664 | | | | | (66.3) | | | | 226 | | | | | (55.5) | | | | <0.01 | | | |  |  |  |
| Multiple | | | 2117 | | | | 120 | | | | | (5.5) | | | | 459 | | | | | (10.8) | | | | 1357 | | | | | (33.7) | | | | 181 | | | | | (44.5) | | | | <0.01 | | | |  |  |  |
| Armed perpetrator(s) | | *Total* | *10637* | | | | *2091* | | | | |  | | | | *4171* | | | | |  | | | | *3984* | | | | |  | | | | *391* | | | | |  | | | |  | | | |  |  |  |
| Yes | | | 2827 | | | | 82 | | | | | (3.9) | | | | 607 | | | | | (14.6) | | | | 1856 | | | | | (46.6) | | | | 282 | | | | | (72.1) | | | | <0.01 | | | |  |  |  |

MCH: Mother and Child Health; SV: Sexual Violence

* For categorical variables, each category was compared with all the other ones

** Up to four recorded associated violence per case
