# Supplementary material for: Assault and care characteristics of victims of sexual violence in eleven Médecins Sans Frontières programs in Africa. What about men and boys?
Source: PLoS One. 2020 Aug 4;15(8):e0237060. doi: 10.1371/journal.pone.0237060 (PMC7402504; doi:10.1371/journal.pone.0237060)
Supplement: S2 Table — (DOCX) [file pone.0237060.s002.docx]

**S2 Table. Differences in access to care and treatment received between age categories among female patients in eleven MSF programs in Africa, 2011 to 2017**

|  | | | | Total | Children  (0-12y) n (%) | | | Adolescents (13-19y)  n (%) | | | Young adults (20-45y)  n (%) | | | Older adults (>45y)  n (%) | | | Cuzick^*^ trend test p-value |
| --- | --- | --- | --- | --- | --- | --- | --- | --- | --- | --- | --- | --- | --- | --- | --- | --- | --- |
| SV clinic setup | *Total* | | | *12508* | *2342* | |  | *4570* | |  | *5116* |  | | *480* | |  |  |
| Integrated into MCH unit | | | | 3045 | 276 | | (11.8) | 970 | | (21.2) | 1590 | (31.1) | | 209 | | (43.5) | <0.01 |
| Standalone | | | | 7579 | 2010 | | (85.8) | 3342 | | (73.1) | 2041 | (39.9) | | 186 | | (38.8) | <0.01 |
| Integrated into care for violence victims | | | | 1884 | 56 | | (2.4) | 258 | | (5.7) | 1485 | (29.0) | | 85 | | (17.7) | <0.01 |
| Time to presentation for care | | | *Total* | *12365* | *2238* | |  | *4552* | |  | *5097* |  | | *478* | |  |  |
| Less than 72 hours | | | | 5727 | 1071 | | (47.9) | 1906 | | (41.9) | 2577 | (50.6) | | 173 | | (36.2) | 0.06 |
| 72 hours – 1 month | | | | 2480 | 603 | | (26.9) | 1135 | | (24.9) | 682 | (13.4) | | 60 | | (12.5) | <0.01 |
| 1 month and above | | | | 4158 | 564 | | (25.2) | 1511 | | (33.2) | 1838 | (36.0) | | 245 | | (51.3) | <0.01 |
| Reason for delay** | | *Total* | | *11418* | *2004* | |  | *4118* | |  | *4835* |  | | *461* | |  |  |
| No delay | | | | 5727 | 1071 | | (53.4) | 1906 | | (46.3) | 2577 | (53.3) | | 173 | | (37.5) | 0.48 |
| No access to healthcare | | | | 1447 | 356 | | (17.8) | 485 | | (11.8) | 541 | (11.2) | | 65 | | (14.1) | <0.01 |
| No knowledge about treatment | | | | 1707 | 95 | | (4.7) | 389 | | (9.4) | 1075 | (22.2) | | 148 | | (32.1) | <0.01 |
| Afraid/Ashamed | | | | 1691 | 343 | | (17.1) | 911 | | (22.1) | 395 | (8.2) | | 42 | | (9.1) | <0.01 |
| Other | | | | 846 | 139 | | (7.0) | 427 | | (10.4) | 247 | (5.1) | | 33 | | (7.2) | <0.01 |
| Total 1^st^ visit package of care provided** | | *Total* | | *11625* | *2134* | |  | *4323* | |  | *4730* |  | | *438* | |  |  |
| Yes | | | | 4206 | 375 | | (17.6) | 1440 | | (33.3) | 2129 | (45.0) | | 262 | | (59.8) | <0.01 |
| Follow up | | | *Total* | *12281* | *2317* |  | | *4505* |  | | *4993* | |  | *466* |  | |  |
| At least one follow-up visit | | | | 6952 | 1176 | (50.8) | | 2325 | (51.6) | | 3118 | | (62.5) | 333 | (71.5) | | <0.01 |
| Mean number of follow-up visits | | | | 1.0 | 0.9 |  | | 0.9 |  | | 1.2 | |  | 1.2 |  | | <0.01^$^ |

MCH: Mother and Child Health; SV: Sexual Violence

* For categorical variables, each category was compared with all the other ones

** Up to four recorded associated violence per patient

^$^ Kruskal-Wallis test for equality of distribution
