# Supplementary material for: Assault and care characteristics of victims of sexual violence in eleven Médecins Sans Frontières programs in Africa. What about men and boys?
Source: PLoS One. 2020 Aug 4;15(8):e0237060. doi: 10.1371/journal.pone.0237060 (PMC7402504; doi:10.1371/journal.pone.0237060)
Supplement: S3 Table — This checklist was elaborated using formal items recommended for cross-sectional studies from STROBE statement (https://www.strobe-statement.org). (DOC) [file pone.0237060.s003.doc]

**S3 Table. Completed STROBE checklist for cross-sectional studies and analysis plan information**

This checklist was elaborated using formal items recommended for cross-sectional studies from STROBE statement (https://www.strobe-statement.org).

|  | Item No | Recommendation | Respected ? | Comments and quotes | |
| --- | --- | --- | --- | --- | --- |
| **Title and abstract** | 1 | (*a*) Indicate the study’s design with a commonly used term in the title or the abstract | Yes | | Study design is indicated in the Methods/Findings section of the abstract  “This was a multi-centric cross-sectional study using routine program data.” Page 2 L38 |
|  |  | (*b*) Provide in the abstract an informative and balanced summary of what was done and what was found | Yes | | These information are stated in the study abstract (study objective described, method and results described) |
| Introduction | | |  | |  |
| Background/rationale | 2 | Explain the scientific background and rationale for the investigation being reported | Yes | | Rationale and existing literature are stated in the introduction section |
| Objectives | 3 | State specific objectives, including any prespecified hypotheses | Yes | | A statement at the end of the introduction specifies the specific goals and objectives.  “The aims of this study were two-fold:  1) To document assault-, perpetrator-, and victim-related characteristics of male-directed as compared to female-directed SV in the specified MSF SV programs. Specifically, we assessed the characteristics of male victims presenting for care across different assault types, circumstances, and perpetrator profiles, in different political contexts as compared to female victims and by age categories.  2) To document characteristics of presentation to care of male and female victims of SV in the specified MSF programs. Specifically, we assessed differences between male and female SV experiences according to context, SV program setup, presentation for care, and treatment received, in the different age categories.” Page 6 L110 |
| Methods | | |  | |  |
| Study design | 4 | Present key elements of study design early in the paper | Yes | | Study design is stated in the first subsection of Methods. Key elements are all described in the methods.  “This was a cross-sectional study using routine multi-centric program data.” Page 7 L123 |
| Setting | 5 | Describe the setting, locations, and relevant dates, including periods of recruitment, exposure, follow-up, and data collection | Mostly | | Setting, contexts, dates of inclusion, are fully described in the method section under “Setting” and “Study population” headlines page 7 and 8.  Locations of the programs included could not be disclosed due to security issues linked to the sensitivity of the data in some countries. |
| Participants | 6 | (*a*) Give the eligibility criteria, and the sources and methods of selection of participants | Yes | | Study population is described is the method section (Study population headline), as well as selection criteria  “All cases recorded in the eleven MSF SV programs of interest between 2011 and 2017 presenting after any kind of SV were included in the study. Suspect cases, as well as those with gender information missing, were not included.” Page 8 L149 |
| Variables | 7 | Clearly define all outcomes, exposures, predictors, potential confounders, and effect modifiers. Give diagnostic criteria, if applicable | Yes | | Standardized variable definitions were used across all programs, which are presented in method section. Validity of all variables was previously assessed for misunderstanding and potential cofounders, and the ones with clear problems were not used in any part of the analysis  “Box 1: Definitions of program variables collected in MSF SV programs in Africa, as per MSF OCB operational protocol, 2011 to 2017” page 9 L184  “Box 2: Definitions of patient variables collected in MSF SV programs in Africa, 2011 to 2017” page 10 L189 |
| Data sources/ measurement | 8* | For each variable of interest, give sources of data and details of methods of assessment (measurement). Describe comparability of assessment methods if there is more than one group | Yes | | Data collection and measurement was the same for all variables, and is described in the methods section. Data came from different programs, but were pooled and cleaned at global level.  “We used the standardized SV database routinely implemented in all SV programs of MSF OCB. […] Data collection was conducted at field level by medical staff using a paper-based patient data form. […] Data were then pooled, validated and anonymized at headquarter level for compilation of the master study database.” Page 9 L169 |
| Bias | 9 | Describe any efforts to address potential sources of bias | Yes | | We notably tried to reduce bias by excluding suspect cases. The analysis section also explains part of our analysis was adjusted on type on context in order to reduce bias expected from this part.  “Crude odds ratio (OR) and adjusted odds ratio analysis controlling for type of context (urban stable zone, conflict zone, post-conflict zone, migratory zone) were calculated” page 11 L197 |
| Study size | 10 | Explain how the study size was arrived at | Yes | | The method describes the inclusion of different programs over several years. The sample is further described in Table 1 |
| Quantitative variables | 11 | Explain how quantitative variables were handled in the analyses. If applicable, describe which groupings were chosen and why | Yes | | Definitions of all categories for variables are presented in Box 1 and 2.  “Box 1: Definitions of program variables collected in MSF SV programs in Africa, as per MSF OCB operational protocol, 2011 to 2017” page 9 L184  “Box 2: Definitions of patient variables collected in MSF SV programs in Africa, 2011 to 2017” page 10 L189 |
| Statistical methods | 12 | (*a*) Describe all statistical methods, including those used to control for confounding | Yes | | These are described in the method section.  “The data from the eleven programs, already available in Excel or EpiData software, were pooled and statistical analysis was performed using Stata v13. Missing values were not inferred. Descriptive analysis of data was conducted, and differences between groups were assessed using Pearson’s χ2 test (Chi-square). Crude odds ratio (OR) and adjusted odds ratio analysis controlling for type of context (urban stable zone, conflict zone, post-conflict zone, migratory zone) were calculated. The level of significance was set at α = 5% and 95% confidence intervals (CI) were calculated. Cuzick’s test was used to test for trends across age categories [27], and the Kruskal-Wallis test for equality of distribution [28].” page 10 L194 |
|  |  | (*b*) Describe any methods used to examine subgroups and interactions | Yes | | This is described in the method section. Interaction examination was not applicable. |
|  |  | (*c*) Explain how missing data were addressed | Yes | | This is described in the method section.  “Missing values were not inferred” page 10 L195 |
|  |  | (*d*) If applicable, describe analytical methods taking account of sampling strategy | N/A | | Non applicable |
|  |  | (*e*) Describe any sensitivity analyses | N/A | | Non applicable |
| Results | | |  | |  |
| Participants | 13* | (a) Report numbers of individuals at each stage of study—eg numbers potentially eligible, examined for eligibility, confirmed eligible, included in the study, completing follow-up, and analysed | Yes | | This is described at the beginning of result section  “There were 16715 cases recorded in the database and treated in MSF SV clinics. Of these, 3031 were excluded due to unclear SV event (unknown type of event n=408, non-sexual aggression n=640, suspect n=1983), and 134 were excluded as gender information was not recorded.” Page 12 L212 |
|  |  | (b) Give reasons for non-participation at each stage | Yes | | This is described at the beginning of result section.  “There were 16715 cases recorded in the database and treated in MSF SV clinics. Of these, 3031 were excluded due to unclear SV event (unknown type of event n=408, non-sexual aggression n=640, suspect n=1983), and 134 were excluded as gender information was not recorded.” Page 12 L212 |
|  |  | (c) Consider use of a flow diagram | N/A | | Use of a flow diagram was not deemed appropriate |
| Descriptive data | 14* | (a) Give characteristics of study participants (eg demographic, clinical, social) and information on exposures and potential confounders | Yes | | Table 1 describes the participants and programs included.  “Table 1: Characteristics of eleven MSF sexual violence programs in Africa, 2011 to 2017” page 12 L222 |
|  |  | (b) Indicate number of participants with missing data for each variable of interest | Yes | | The total numbers of recorded data for each variable are stated in variable headline of each table |
| Outcome data | 15* | Report numbers of outcome events or summary measures | Yes | | All numbers are reported in Tables |
| Main results | 16 | (*a*) Give unadjusted estimates and, if applicable, confounder-adjusted estimates and their precision (eg, 95% confidence interval). Make clear which confounders were adjusted for and why they were included | Mostly | | All adjusted estimates, and 95% confidence intervals, are reported in Table 2. Non-adjusted estimates were not displayed in the interest of table clarity. However, the effect of adjustment on significance of all odds ratios is described in the table with the use of footnotes. |
|  |  | (*b*) Report category boundaries when continuous variables were categorized | Yes | | Category boundaries are displayed in variable headings in the tables, where applicable (age groups) |
|  |  | (*c*) If relevant, consider translating estimates of relative risk into absolute risk for a meaningful time period | N/A | | N/A |
| Other analyses | 17 | Report other analyses done—eg analyses of subgroups and interactions, and sensitivity analyses | N/A | | N/A |
| Discussion | | |  | |  |
| Key results | 18 | Summarise key results with reference to study objectives | Yes | | Key results are described at the beginning of discussion section (page 20), and later on by the mean of a paragraph displaying main operational implications (page 26). They also are summarized in the conclusion |
| Limitations | 19 | Discuss limitations of the study, taking into account sources of potential bias or imprecision. Discuss both direction and magnitude of any potential bias | Yes | | Description of limitations is done under “Strengths and limitations” heading in the discussion (page 25). |
| Interpretation | 20 | Give a cautious overall interpretation of results considering objectives, limitations, multiplicity of analyses, results from similar studies, and other relevant evidence | Yes | | References were added where possible, and discussed. Limitations were taken into account in the discussion. |
| Generalisability | 21 | Discuss the generalisability (external validity) of the study results | Yes | | Study results were deemed generalizable at African level, and discussed as such. |
| Other information | | |  | |  |
| Funding | 22 | Give the source of funding and the role of the funders for the present study and, if applicable, for the original study on which the present article is based | Yes | | Funding information were displayed upon submission but not included in the manuscript, as requested. |
| **Additional information on analysis plan** | | |  | |  |
| “For observational studies, authors are required to clearly specify (a) What specific hypotheses the researchers intended to test, and the analytical methods by which they planned to test them; (b) What analyses they actually performed; and (c) When reported analyses differ from those that were planned, authors must provide transparent explanations for differences that affect the reliability of the study's results.” PLoS Med submission guidelines | | | (a) The goal of this study is stated page 6 L110. Analysis was first planned to include odds ratio calculation for male-female comparison, adjusted on type of context, for variables describing access to care  (b) Odds ratio analysis was fully performed as planned. In addition, trend analysis among age groups was performed, given the clearly different patterns of accessing care found in the different stages of life  (c) The reliability of the study results was not affected | | |
| “If a prospective analysis plan (from the study's funding proposal, IRB or other ethics committee submission, study protocol, or other planning document written before analyzing the data) was used in designing an observational study, authors must include the relevant prospectively written document with the manuscript submission for access by editors and reviewers and eventual publication alongside the accepted paper. If no prospectively written document exists, authors should explain how and when they determined the analyses being reported.” PLoS Med submission guidelines | | | No formal protocol relative to this study is available; MSF internal policy does not allow for non-formal study document sharing.  Analyses were decided at project initiation in accordance with all authors of the study, and then revised following database building and appearance of clear sexual violence patterns according to age groups. | | |

*Give information separately for exposed and unexposed groups.
